# Iron Complexation to Oxygen Rich Marine Natural Products: A Computational Study

**DOI:** 10.3390/md8010001

**Published:** 2010-01-04

**Authors:** Thomas J. Manning, Jimmy Williams, Joey Jarrard, Teresa Gorman

**Affiliations:** Chemistry Department, Valdosta State University, Valdosta, GA 31698, USA; E-Mails: jiwilliams@valdosta.edu (J.W.); hoit_04@hotmail.com (J.J.); thg0001@auburn.edu (T.G.)

**Keywords:** kahalalide F, halichondrin B, discodermolide, marine natural product, aqueous stability factor, computational

## Abstract

The natural products kahalalide F, halichondrin B, and discodermolide are relatively large structures that were originally harvested from marine organisms. They are oxygen rich structures that, to varying degrees, should have the ability to bind iron (II or III) by Fe-O and/or Fe-N bonds. In this semi empirical study, the binding of these natural products to iron (II) is studied and the aqueous stability factor (ASF) is used to determine which bonding configuration is most stable. The energy, the complex charge (+1), the average Fe-O (or Fe-N) bond distances and the dipole moments are used to calculate the ASF. The ASF provides insight to which complex will be the most stable and water soluble, important for a medicinal application. The ability of a molecule with a more than six oxygen and/or nitrogen atoms to bind iron (hexavalent, octahedral) by shifting which six atoms (O/N) are bound to the iron qualifies it as a polarity adaptive molecule.

## 1. Introduction

**Figure 1:**
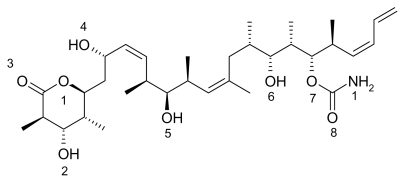
The two dimensional image of the marine natural product discodermolide.

Past work in this lab with marine natural products (MNPs) has focused on experimental aspects associated with the marine natural product bryostatin, which is extracted from the bryozoa, *Bugula neritina*, and the pharmaceutical agent ET743 (Yondelis^™^) extracted from the sea squirt, *Ecteinascidia turbinate*. It is accepted that symbiotic microbes produce bryostatin and that this substance may play a role in the defense of the host organism and/or be a siderophore. Our geochemical studies helped understand the chemical environment under which the symbiotic microbes that produce the marine natural products can thrive. This work led us to develop an approach to producing marine natural products called pharmaceutical aquaculture which is centered on a device called bacterial amplification chambers (BACs). During these projects we found that bryostatin was often bound to iron when it was extracted from the marine environment. This led us to postulate that bryostatin’s role in nature is that of the siderophore. Bryostatins structure features a bryophan ring lined by a number of oxygen atoms and suggests it has the ability to trap a cation, much like a siderophore or a crown ether. Our group has performed a number of semi-empirical computational studies aimed at determining if iron (III) or iron (II) could bind bryrostatin *via* an octahedral, hexavalent type geometry. This work was than extended to examining bryostatin analogs, some well known siderophores, some other natural products and taxol. From a pharmaceutical perspective, it was argued that iron binding to many of these natural products increased its water solubility, stabilized its structures and made the complex more rigid, perhaps allowing it to perform in a lock and key model more efficiently. In this computational study we are focused on the marine natural products kahalalide F, halichondrin B, and discodermolide. Discodermolide (Figure 1) is part of a class of anticancer drugs that target microtubules and has been shown to stimulate microtubule polymerization and stabilize microtubules at high concentrations similar to that of taxol [1,2].

Discodermolide has better water solubility parameters and higher activity against some taxol-resistant cell lines. Discodermolide was first extracted from the deep-water, Caribbean sponge, *Discodermia dissolute*, in 1990 [3]. It has been tested in different *in vivo* and *in vitro* experiments and advanced to Phase I clinical trials [4–6]. A number of analogues of discodermolide have been synthesized, but maintain the carbon backbone [7].

**Figure 2:**
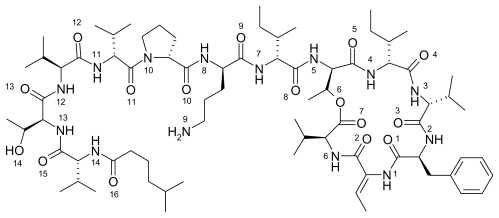
The two dimensional structure of the marine natural product kahalalide F. Oxygen atoms are numbered 1–16 and nitrogen’s are numbered 1–14.

Kahalalide F is a marine natural product that belongs to a family of compounds known as depsipeptides (see Figure 2) [8–13]. It is most commonly obtained from the Hawaiian saltwater mollusk, *Elysia rufescens* [8]. Kahalalide F has also been isolated from the green algae, *Bryopsis pennat*, which is part of *E. rufescens* diet. This finding suggests that kahalalide F is a secondary metabolite derived from the mollusk’s diet [9]. The structure of kahalalide F was first described by Hamann *et al.*; however, the stereochemistry of its more active form was later elucidated by Rinehart *et al.* [13]. Kahalalide F has been tested in multiple clinical trials and has been found effective against many human cancers, including prostate, breast, colon carcinomas, neuroblastoma, chondrosarcoma, osteosarcoma, non-small-cell lung cancer, liver cancer, and melanoma. It has been shown to attack tumor cells *via* multiple mechanisms including; disruption of lysosomal membranes, inhibition of transforming factor-α expression, blockage of epidermal growth factor signaling pathways, and induction of non-p53 mediated apoptosis. Kahalalide F has been in clinical trials including patients with androgen refractory prostate cancer and advanced solid tumors [14–16].

**Figure 3:**
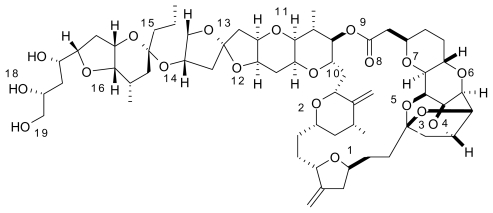
Halichondrin B has proven to be a difficult molecule to synthesize on a large, economical scale.

**Figure 4:**
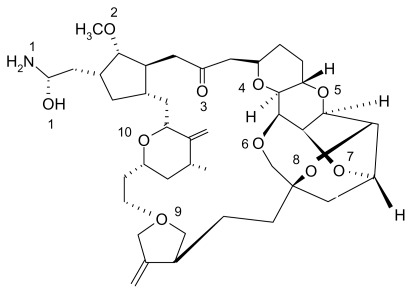
A two dimensional structure of E7389.

Halichondrin B was isolated from the Japanese sponge *Halichondria Okada* in 1985 by Uemura *et al.* [17]. Halichondrin B is a powerful polyether macrolide (see Figure 3). Since 1986, halichondrin B and its analogs have been found in several different sponges, but with a very low yield [18]. The National Cancer Institute has been interested in developing halichondrin B for preclinical trials, but has not followed through because harvesting and extraction efforts produced low yields. Halichondrin B has been shown to be a strong anticancer agent [19] especially in the treatment of leukemia and reducing tumors in other cancers including lung, pancreatic, and ovarian cancer. The first synthesis was completed by Namba *et al*. [20] which eventually led to the discovery of the halichondrin B analog, E7389 [21]. E7389 (Figure 4) has been proven to be more stable than halichondrin B and will be discussed next. It is a synthetic analog of halichondrin B and acts as a microtubule modulator [22]. The total synthesis of E7389 has been accomplished and it has strong medicinal properties [23–25].

In the figures above (1–3) the oxygen and nitrogen atoms are numbered and will be correlated with data presented below. Like many marine natural products, these structures are relatively large and difficult to synthesize and there has been no success in getting marine microbes to synthesize the three natural products in bulk. A key point to recognize is that the iron ion can move to different Fe-O and/or Fe-N bonds in the aqueous phase. By shifting from one Fe-O bond to another Fe-O bond the molecule shifts polarity and geometry. This ability to shift charge and shape allows the iron complex to adapt to different environments in a physiological environment. For example, a complex with a high dipole moment will have a higher solubility in water. If the iron shifts the specific oxygen atoms to which it is bound, the polarity can be lowered and will be more likely to penetrate a nonpolar cell wall. From our past work, we have dubbed this a polarity adaptive molecule. The reason to investigate the binding of these different molecules to iron is because it may enhance their medicinal activity.

## 2. Results and Discussion

The aqueous stability factor (ASF, Joules*meters/Debye or Jm/D)) is a term that combines four calculated parameters for the different metallic-marine natural product complexes; complex energy (E), average Fe-O bond length (L), charge (Z, +1, +2, *etc*.) and the dipole moment (D).

(1) $$\begin{array}{l} \text{ASF}=(\text{Complex Energy})(\text{Average Bond Length})/(\text{Dipole Moment})(\text{Charge})\\ =({\text{E}}^{*}\text{L}/{\text{D}}^{*}\text{Z}) \end{array}$$

For a molecule to be stable, the calculated magnitude of the energy (E) should be small or negative. For strong Fe-O bonds, a small Fe-O average bond length indicates a great degree of covalency. In narrowing down the number of complexes, compounds with average bond distances of more than 2.7 Å were discarded without considering other parameters and any single bond of more than 2.9 Å was considered to be close to lacking covalency and that complex was also not considered. For a molecule to have good water solubility, a desirable medicinal characteristic, a large dipole moment (D) is desired. Also, larger charges (*i.e.*, +2, +3) would improve water solubility but our mass spectral data (*via* FT-ICR, TOF-MS) on complexes like Fe-bryostatin-1 and Fe-taxol only suggested the +1 species was present. The smaller the aqueous stability factor, the more likely the complex is to have a physiological solubility and improved stability *in vivo*.

**Figure 5:**
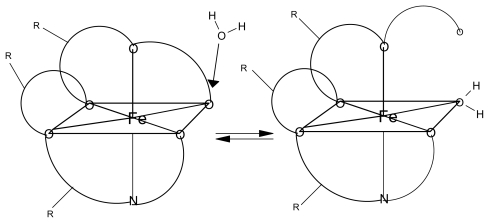
Iron has an octahedral geometry with six coordination sites. (left) In some calculations all six sites are occupied by oxygen and/or nitrogen atoms that are part of the marine natural product. In the other calculations (right) five of the six sites are occupied by oxygen and/or nitrogen atoms from the marine natural product and the sixth site is occupied by a water (solvent) molecule.

While the dipole moment is used to calculate the ASF, it is also used to help understand the solubility of the solute in a common solvent (water, methanol, *etc*.). With the common solvents shown in Table 1, the dipole moment to volume ratio (D/V) extends from the polar solvent water (.090) to the nonpolar species hexane (0.00). Tables 2–5 provide results from this study for six Fe-O bonds where all six of the oxygen’s are part of the marine natural product and Tables 6–9 provide the computational results for complexes with five Fe-O bonds and one Fe-OH_2_ bond (see Figure 5). The iron still has an octahedral geometry with a hexavalent bonding scheme but experimental work with Fe-taxol and Fe-bryostatin have shown that a water molecule can occupy one of the six coordination sites. The numbers in the first column of Tables 2–9 (*i.e.*, 6, 7, 8, 9, 10, 11) are correlated with the oxygen and/or nitrogen atoms numbered in Figures 1–4. The average bond distance (column 3) is for the six Fe-O bond lengths that formed the hexavalent complex.

Tables 2, 3, 4, 5, 6 and 7 provide the calculated parameters for the most stable iron-kahalalide F, iron-halichondrin B and iron-discodermolide complexes. Over two-hundred complexes were built and studied for selection into these tables; those selected for these tables had the most stable ASF calculations. In table 2, 3, and 4 iron is taken as a hexavalent central atom with an octahedral geometry and all six oxygen and/or nitrogen atoms (6 Fe-O/Fe-N total) are from the MNP structure. In Tables 5, 6 and 7 iron has the same geometric features but only five of the oxygen’s and/or nitrogen’s from the MNP are part of complexes inner sphere while the sixth coordination spot on the central atom is occupied by a water molecule (Fe-OH_2_).

**Figure 6:**
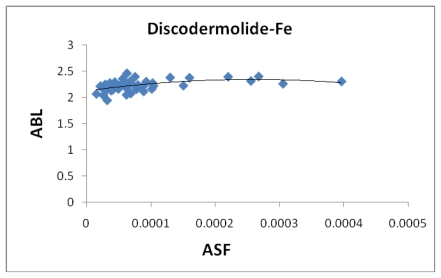
The average bond length (Å) plotted against the aqueous stability factor (Jm/D) for discodermolide bound to iron only. The best fit equation is a polynomial *y* = −3*10^6^*x*^2^ + 1693.9*x* + 2.1305 which gave a correlation coefficient of *R*^2^ = 0.2543.

Figure 6 shows the correlation between the average bond length (ABL) in the iron-Discodermolide (Fe-Dis) complex and the calculated ASF. While there is not a strong correlation, short Fe-O and Fe-N bonds are critical for a strong metallic complex. These bonds are also dynamic in that the Fe-O and Fe-N bonds are constantly breaking and forming. The fifty most favorable (lowest ASF) complexes are presented in the graph and the individual values for the ten most favorable complexes are given in table 2. Tables two through seven also provide the individual bond distances for the ten most favorable complexes. Figure 6 is indicative of the three complexes regarding the lack of impact of the ABL on the ASF’s for the complexes studied here. While the ASF values can vary up to an order of magnitude for the different complexes, the ABL values are typically within 10–15% of each other.

**Figure 7:**
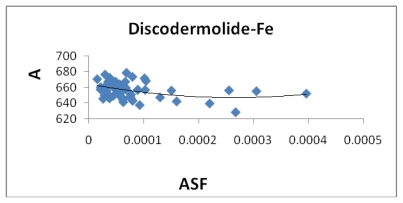
The area (Å^2^) plotted against the aqueous stability factor (Jm/D) for discodermolide bound to only iron. The best fit equation is a polynomial *y* = 3*10^8^*x*^2^ − 134702*x* + 664.51 and a correlation coefficient of *R*^2^ = 0.1468.

**Figure 8:**
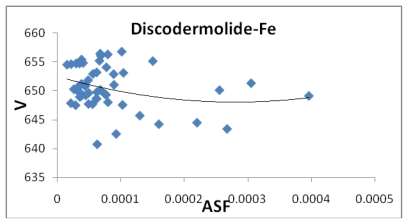
The volume (Å^3^) plotted against the aqueous stability factor (Jm/D) for discodermolide bound to iron only. The best fit equation for the data set is the polynomial *y* = 6*10^7^*x*^2^ − 31647*x* + 652.51 (*R*^2^ = 0.0749).

Figures 7, 8, 13, and 14 illustrate the poor correlation between the complexes surface areas and volumes and the ASF for the Fe-Dis and Fe-Dis-H_2_O. Although not presented, other complexes show a similar poor correlation for volumes and surface areas plotted against the ASF. While these graphs indicate no significant impact of the volume or surface area on the ASF, it may play a role in the ASF and the Dipole Moment/Volume (D/V) ratios. Given that any natural product can interact with a protein, DNA, RNA, cell wall, *etc*., shifts in its geometry should be considered an important parameter in medicinal applications. Although not understood now, correlating these parameters with its medicinal activity as well as side effects may be explained in the future. As the ratio of area to the volume (A/V) approaches one, the ASF gets smaller suggesting a complex with the same surface area and volume would be the ideal candidate for water solubility and stability. While the volumes of the fifty most stable complexes vary by approximately 17 Å^3^, the volumes of the ten most stable complexes vary over a smaller range (5 Å^3^).

**Figure 9:**
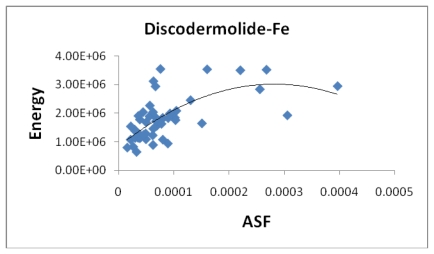
The energy (joules/mol) plotted against the aqueous stability factor (Jm/D) for discodermolide bound to iron only. The best fit equation for this correlation is the polynomial *y* = −3*10^13^*x*^2^ + 2*E*+10*x* + 838341 (*R*^2^ = 0.4491).

**Figure 10:**
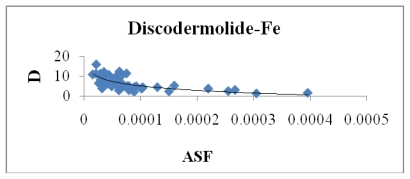
The dipole moment (Debye) plotted against the aqueous stability factor (Jm/D) for discodermolide bound to iron only. The best fit equation for the data set is the polynomial *y* = −3.177ln(*x*) − 24.188 with a correlation coefficient of *R*^2^ = 0.4878.

**Figure 11:**
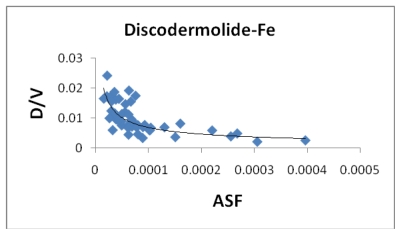
The dipole moment to volume ratio (D/Ǻ^3^) plotted against the aqueous stability factor (Jm/D) for discodermolide bound to iron only. The best fit equation is *y* = 3*10^−05^*x*^−0.576^ (*R*^2^ = 0.5859).

Figure 8 indicates a small correlation between the calculated dipole moment of the Fe-Dis complexes and the ASF. This should be expected since increasing the dipole moment increases its water solubility. Figure 9 illustrates a modest correlation between the complexes energy and the ASF. High energy values were eliminated by setting upper limits on Fe-O and Fe-N bond distances considered (longer bond distances = higher energies and lower stabilities). Figures 10 and 11 illustrates the correlation between the dipole moment and the ASF, and the D/V (dipole moment/volume) ratio and the ASF, respectfully. While the D/V ratio is a better indicator of solubility in a specific solvent, the dipole moment of the different Fe-Dis complexes is used to calculate the ASF (Equation 1). Despite this dependency, the D/V ratio shows a better correlation verses the ASF than does the dipole moment (0.4878 *vs.* 0.5859).

**Figure 12:**
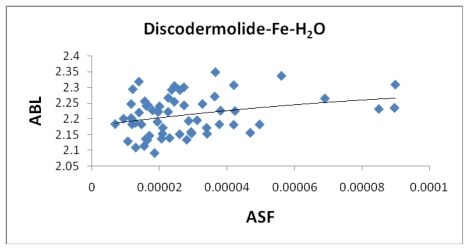
The average bond length (Å) plotted against the aqueous stability factor (Jm/D) for the Fe-Dis-H_2_O complex. The best fit line was determined to be the polynomial *y* = −5*10^6^*x*^2^ + 1408*x* + 2.1771 (*R*^2^ = 0.0867).

**Figure 13:**
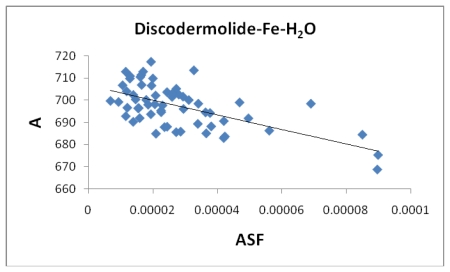
The area (Ǻ^2^) plotted against the aqueous stability factor (Jm/D) for the Fe-Dis-H_2_O complex. The best fit line is *y* = 706.59e^−475^*^x^* (*R*^2^ = 0.3831).

**Figure 14:**
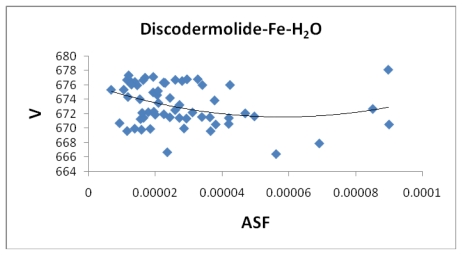
The volume (Å^3^) plotted against the aqueous stability factor (Jm/D) for the Fe-Dis-H_2_O complex. The best fit equation was determined to be *y* = 1*10^9^*x*^2^ − 154248*x* + 676.07 (*R*^2^ = 0.0943).

**Figure 15:**
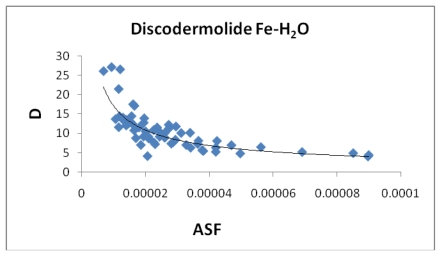
The dipole moment (Debye) plotted against the aqueous stability factor (Jm/D) for the Fe-Dis-H_2_O complex. The best fit line for this data set is *y* = 0.0085*x*^−0.661^ with a correlation coefficient of *R*^2^ = 0.6993.

**Figure 16:**
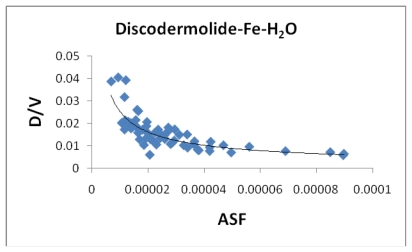
The dipole moment to volume ratio (D/Ǻ^3^) plotted against the aqueous stability factor (Jm/D) for the Fe-Dis-H_2_O complex. The best fit equation for this data set is *y* = 1*10^−05^*x*^−0.659^ (*R*^2^ = 0.6974).

Table 5 provides the ten most favorable complexes for the iron-discodermolide-water (Fe-Dis-H_2_O) compound. Figure 12 provides the correlation between the average bond lengths for 50 of the water containing complexes. The average bond length over all 50 complexes is slightly longer than the average bond length for the 50 complexes with no water (2.17 Å *vs*. 2.13 Å) and, while there is a general range the general bond distances and ASF fall within, there is no correlation between the two parameters. The average surface area (Figure 13) increases from the Fe-Dis to the Fe-Dis-H_2_O complex from an average of 664 Å^2^ to 707 Å^2^. This increase in surface area is similar to that of an individual water molecule (36.4 Å^2^). The average volume (Figure 14) for the fifty complexes (676 Å^3^) for the water containing complex is larger than the dehydrated complex (652 Å^3^) by an amount that is similar to the volume of the water molecule (19.4 Å^3^). The average area for the fifty water containing complexes varies by as much as 50 Å^2^ while the volume parameters are all within 12 Å^3^ of each other which suggests the different complexes should have similar densities but the different surface areas may lend themselves to different levels of activities. Figure 15 illustrates a strong correlation between the calculated dipole moment and the ASF and Figure 16 shows a slightly stronger correlation with the D/V ratio for the Fe-Dis-H_2_O complexes. The higher D/V ratio’s represent more polar complexes. A value in the 0.04–0.045 range should be soluble in a solvent such as ethanol or methanol with some solubility in water. The other end of the D/V scale is 0.005 which would indicate solubility in a low polarity solvent (*i.e.*, octanol) or a nonpolar solvent (*i.e.*, hexane). This range of values indicates that the Fe-Dis-H_2_O complex is a polarity adaptive molecule.

**Figure 17:**
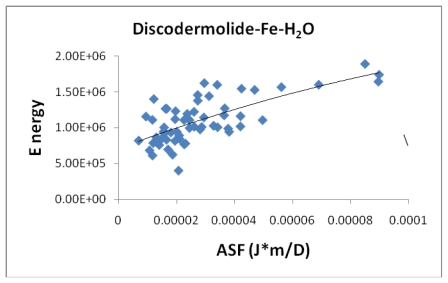
The energy (joules) plotted against the aqueous stability factor (Jm/D) for the Fe-Dis-H_2_O complex. The best fit equation was the polynomial *y* = −4*10^13^*x*^2^ + 2*E*+10*x* + 704724 (*R*^2^ = 0.4903).

Figure 17 shows a modest correlation between the energy and the ASF. The average energy for the Fe-Dis-H_2_O complexes are lower than the Fe-Dis complex (704.7 kJ *vs*. 838.8 kJ) indicating that the solvent will enter the inner sphere of the octahedral complex and stabilize it. Also, the average dipole moment for the fifty Fe-Dis-H_2_O complexes is 13.36 Debye while the average for the fifty Fe-Dis is 8.39 Debye. The range of dipole moments is larger for the Fe-Dis-H_2_O complex compare to the Fe-Dis indicating a wider range of polarities it can adapt to in a physiological environment. The addition of the polar water molecule to the complex results in a significant increase in average polarity and the ASF.

When plotted, the Fe-Hali and Fe-Hali-H_2_O complexes also show little correlation for the same paramters. For the average dipole moments and correlation coefficients of the Fe-Kah (15.99 D, 0.4697) and Fe-Kah-H_2_O (19.04 D, 0.0626) show an increase in the average calculated dipole moment for the water containing complex. The average D/V ratio, which is a better measure of water solubility than just the dipole moment for larger complexes, for the Fe-Kah (0.0103 D/Å^3^) is slightly lower than the D/V for the Fe-Kah-H_2_O (0.0121 D/Å3)) but both have similar or improved correlation verses the ASF compared to just the dipole moment. The average energy for the fifty Fe-Kah (2.005 MJ) is higher than the fifty Fe-Kah-H_2_O complexes (1.55 MJ) indicating that, on average, these complexes are less than when dehydrated. In both groups of complexes the correlation between the energy and the ASF is more significant than most of the other parameters discussed.

**Figure 18:**
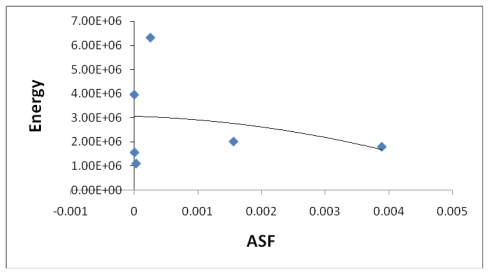
Using the data in table 8, the average values for the ASF for the fifty more stable complexes of the different structure (Fe-Hali, Fe-Hali-H_2_O, *etc*.) is plotted against their energy (x-axis, J) and gives essentially no correlation represented by the best fit polynomial *y* = −7*E*+10*x*^2^ − 8E+07*x* + 3*E*+06 (*R*^2^ = 0.0774).

**Figure 19:**
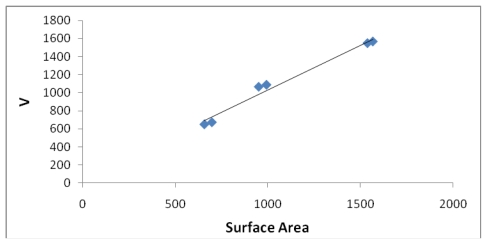
The average ratio of volume to surface area, both calculated in Å, is close to 1.0. The best fit line for the data from table 8 gives a straight line fit of *y* = 0.9934*x* + 38.626 (*R*^2^ = 0.9787).

**Figure 20:**
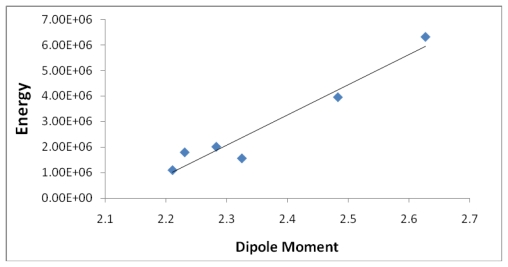
The correlation between the calculated energy and the dipole moment for the six groups of complexes gives a strong correlation. (*y* = 1*E*+07*x* − 3*E*+07, *R*^2^ = 0.9397).

**Figure 21:**
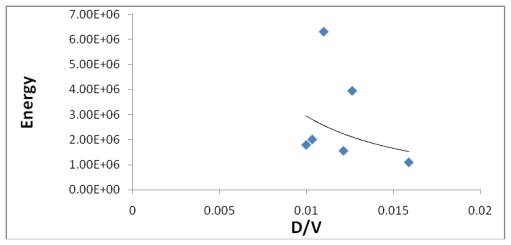
The average energy of the six groups is plotted against the calculated D/V ratio and gives no correlation indicating there is little correlation between solubility and the complexes energy (*y* = 4013*x*^−1.432^, *R*^2^ = 0.1416).

Table 8 and Figures 18, 19 and 20 provide a summary of the average values given for each set of complexes. Figure 18 is a plot of the average energies for the different complexes verses the aqueous stability constant. Figure 19 shows that the average volumes and average surfaces are all closely correlated in that the ratio (V/A) is slightly less than one for most complexes. The energy does show a strong correlation with the complexes dipole moments for the averages of the six groups of iron complexes (Figure 20). On the other hand, the correlation between the energy and the D/V ratio drops significantly (see Figure 21). Overall, the lower energy complex producing a species with a lower dipole moment may not be desirable for medicinal applications. Typically a high dipole moment results in high water stability and low energies means a more stable complex. Reviewing the noncomplexed values (no Fe) in table eight of the three marine natural products shows a lower energy or more stable species–but they also have lower dipole moments and lower D/V ratios than the iron complexes. This lower water solubility justifies the use of iron as a solubility enhancement agent for medicinal applications.

## 3. Materials and Methods

The calculations were performed in Semiempirical (PM3) mode and all Fe-molecule complexes were assigned a charge of +1. The software used in these calculations is the Spartan Linux/cluster version (Wavefunction Inc, Irvine, CA, USA). The SUN Microsystem cluster was the hardware used to calculate the values used in this paper (Tables 1–7). For each molecule, up to one-hundred different Fe-MNP structures (up to fifty with six bonds to molecule, and up to fifty with five bonds to molecule and one to water) were built and calculated. Because of the sheer quantity of structures this study examined we sought to use semi-empirical, as opposed to ab initio, calculations. A single, high accuracy energy value is not considered important but rather the trends of energy values between different complexes. Likewise, a single conformation was not sought for any complex. Because of the dynamic nature of these structures in a physiological environment, a single conformation was never considered as a viable approach to understand their polarity. A well studied analogy is the MRI contrast reagent Gd-DTPA (Gadolinium(III) - diethylenetriaminepentaacetic acid). The exchange rate of water between the inner and outer sphere of the complex occurs on the order of 10^6^ s^−1^ [26]. This involves water displacing a carboxylate on one of Gd(III)’s eight coordination sites.

(2) $${\text{Gd}-\text{DPTA}}^{\text{x}+}(\text{aq})+{\text{H}}_{2}\text{O}(1)\to \text{Gd}({\text{H}}_{2}\text{O}){\text{DTPA}}^{\text{x}+}(\text{aq})$$

Although only projected at this point, all three complexes outlined in this study should be dynamic in both its conformations and how it binds Fe(III) or (II). Conformational energies were evaluated using the Molecular mechanics sybyl force field. A molecule with a large number of single bonds in its backbone, each having three staggered rotamers, will have a large number of conformations. For example, discodermolide with 15 single bonds (fig. 1), can have up to 14,348,907 (3^15^) conformations. Many of these structures will blend together so the actual number of unique conformations will be significantly lower than this value. Energy minimization was used to find the lowest energy structure, assuming a unique Fe-MNP geometry with a hexavalent, octahedral geometry, for each metal-ligand complex. This low energy structure was then used in the semiempirical calculations.

Molecules with smaller numbers of oxygen and/or nitrogen atoms limited the number of configurations. Of these possible one hundred structures for each iron-MNP complex, the ten most stable were selected for the six Fe-MNP and the ten most stable were selected for the five Fe-MNP; one Fe-OH_2_ complexes. Then National Cancer Institutes’ Compare program and data (http://dtp.nci.nih.gov/compare/) was used to obtain the GI_50_ values for halichrodrin.

## 4. Conclusions

This computational project is focused on showing that three well known marine natural products are also polarity adaptive molecules [27–29]. Polarity adaptive molecules are involved in complexes with cations, in this case iron(II), and can shift dipole moments, as well as other parameters (average bond length, area, volume, energy) by shifting which five or six atoms the molecule attaches to iron. Currently we are working on similar calculations with five other molecules (E7389, dolastatin, piperazimycin, hibarimicin, and aplidine). Our ultimate computational exercise will be to attempt to correlate GI_50_ (growth inhibition) values taken from the National Cancer Institute’s five dose, 60 cell line panel and determine if there is a correlation of those values for the various cell lines and the ASF factor.

In a large molecule, dipole moments can take on a different meaning than with a small species such as water or carbon dioxide. In a past study, our group showed that the polarity of a large molecule should be considered in sections when selecting a solvent [30]. In a large molecule a number of conformations are possible and a cation, such as iron (II) can bind it in a number of ways. What this study shows is that depending on conformation and depending on where the cation binds the species, which is a dynamic process, the individual parameters, such as dipole moment, volume, area, bond lengths, *etc*. will vary. With the iron added, the distribution of these values varies over a wider range. Typically molecules are thought of having a specific polarity and subsequently a specific solubility in a certain solvent or a specific solubility in a physiological environment. This work is not intended to focus on a specific molecular geometry or dipole moment but to show that these values are distributed over a range of values. This distribution of values is important as the medicinal agent travels through physiological environment and senses environments with different polarities.

**Figure 22:**
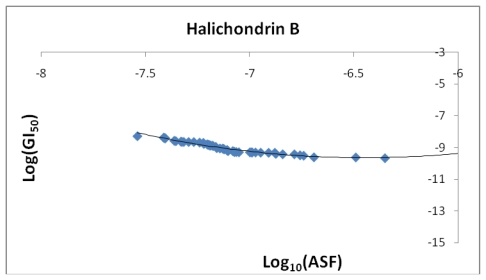
After being sorted by magnitude, the log(GI)_50_ for halichondrin is plotted against the log(ASF). The best fit equation is *y* = 1.3375*x*^2^ + 17.258*x* + 46.007 (*R*^2^ = 0.9509).

Figure 22 shows a projected correlation between the log of the calculated ASF values for the Fe-halichondrin B complexes plotted against the Log(GI_50_) values for halichondrin B verses the National Cancer Institute’s (NCI) 60 cell line test(s). The COMPARE program available through NCI’s DTP web site was used to download the data. The data sets log(ASF) and log(GI_50_) were sorted highest to lowest and plotted against each other. The NCI cell line tests represent a range of cancers (CNS, melanoma, prostate, breast, *etc*.) and different genetic variations within each type of cancer. While the GI_50_ values, which are determined experimentally, and the calculated ASF values, cannot be directly correlated at this stage, we would like to offer the following projection and use Figure 22 as a potential example. First halichodrins medicinal value will be altered if administrated as an iron (II) or iron (III) complex. Second, if uncomplexed halichrodrin B is administrated its ability as a pharmaceutical may be correlated with the concentration of various cations (Fe^3+^, Fe^2+^, Ca^2+^, Zn^2+^, *etc*.) in the cancerous area. The cations can bind and either inhibit or improve the drug’s efficacy. Different genetic variations of the same disease may have different iron levels and subsequently interact with the molecule to different degrees [31].

## Figures and Tables

**Table 1:** The calculated dipole moment (Debye, D), molecular volume (V, Å^3^) and the D/V ratio for some common solvents.

Name	Dipole Moment	Molecular Volume (V)	D/V
**Water**	1.74	19.24	0.090
**Methanol**	1.54	40.66	0.038
**Ethanol**	1.48	59.08	0.025
**1-Propanol**	1.59	77.37	0.020
**1-Butanol**	1.60	95.69	0.017
**1-Pentanol**	1.41	114.06	0.012
**1-Octanol**	1.62	168.95	0.0096
**Hexane**	0.00	124.80	0.00

**Table 2:** The dipole moment (Debye), bond distances (Å), area (Å^2^), volume (Å^3^), dipole/volume ratio (D/Å^3^), molar mass (g/mol), average bond length (Å), energy (joules/mol), and aqueous stability factor (ASF) of ten Discodermolide molecules containing iron.

Oxygen/nitrogen #’s	Dipole	Distances	Area	Volume	D/V	Molar mass	Aver. Bond dist.	Energy	ASF

**O1, O2, O3, O4, O5, O6**	11.17	1.957, 2.485, 1.964, 2.456, 2.184, 2.253	657.4	647.87	0.0172	646.625	2.2165	1073380.93	2.1299 × 10^−5^
**O1, O2, O3, O4, O5, N1**	11.15	1.932, 2.471, 1.978, 2.470, 2.253, 2.396	650.0	647.51	0.0172	646.425	2.2500	1423974.61	2.8734 × 10^−5^
**O1, O2, O3, O4, O8, N1**	8.12	1.900, 2.415, 1.999, 2.412, 2.022, 2.005	676.4	654.77	0.0124	648.641	2.1255	1131756.10	2.9625 × 10^−5^
**O1, O2, O3, O4, O5, O8**	15.76	2.228, 2.444, 1.945, 2.232, 2.208, 2.269	660.4	654.66	0.0241	648.641	2.2210	1538512.45	2.1682 × 10^−5^
**O1, O2, O3, O6, O7, N1**	12.86	2.425, 2.023, 2.015, 1.984, 2.476, 2.160	661.2	649.79	0.0198	647.633	2.1805	1456789.72	2.4701 × 10^−5^
**O1, O2, O3, O5, O8, N1**	7.81	2.068, 2.051, 2.036, 2.106, 1.965, 2.109	656.3	653.23	0.0120	648.641	2.0558	888909.63	2.3400 × 10^−5^
**O1, O3, O4, O6, O8, N1**	9.38	1.899, 1.883, 1.968, 2.148, 1.904, 1.911	655.4	650.65	0.0144	648.641	1.9522	643672.84	1.3396 × 10^−5^
**O1, O3, O4, O7, O8, N1**	15.09	2.181, 2.128, 1.980, 2.439, 2.036, 2.060	667.7	655.45	0.0230	649.649	2.1373	1124527.40	1.5928 × 10^−5^
**O1, O3, O4, O5, O7, O8**	16.44	2.418, 2.353, 2.210, 2.249, 2.427, 2.079	659.5	656.48	0.0250	649.649	2.2893	1723667.00	2.4003 × 10^−5^
**O1, O2, O3, O6, O8, N1**	10.78	2.018, 2.122, 2.027, 2.185, 2.043, 2.049	670.6	654.56	0.0165	648.641	2.0740	795760.82	1.5310 × 10^−5^

**Table 3:** The dipole moment (Debye), bond distances (Å), area (Å^2^), volume (Å^3^), dipole/volume ratio (D/Å^3^), molar mass (g/mol), average bond length (Å), energy (joules), an aqueous stability factor of ten kahalalide F molecules containing iron.

Oxygen/nitrogen #	Dipole	Bond Distances	Area	Volume	D/V	molar mass	aver. bond length	Energy	ASF

**N1, N2, N3,**	18.04	2.499, 2.117, 2.519,	1507.1	1542.8	0.01169	1530.69	2.2373	1.160 × 10^6^	1.44 × 10^−5^
**O5, O6, O7**		2.015, 2.237, 2.037							
**N3, N4, N5,**	22.96	2.409, 2.54, 2.338,	1570.5	1549.5	0.01482	1528.68	2.4077	1.931 × 10^6^	2.03 × 10^−5^
**N6, N7, O1**		2.537, 2.576, 2.046							
**N4, N5, N6,**	20.38	2.472, 2.043, 2.435,	1568.9	1551.6	0.01313	1529.68	2.2800	1.785 × 10^6^	2.00 × 10^−5^
**N7, O1, O2**		2.463, 1.962, 2.305							
**N3, N4, N5,**	25.59	2.478, 2.515, 2.232,	1554.4	1548.7	0.01652	1528.68	2.3548	1.854 × 10^6^	1.71 × 10^−5^
**N6, N7, O2**		2.315, 2.543, 2.046							
**N4, N5, N6,**	23.16	2.492, 2.121, 2.418,	1562.0	1549.3	0.01495	1529.68	2.2795	1.691 × 10^6^	1.66 × 10^−5^
**N7, O2, O3**		2.53, 2.076, 2.04							
**N6, N7, O2,**	23.35	2.499, 2.547, 2.29,	1501.7	1540.1	0.01516	1531.70	2.2952	2.055 × 10^6^	2.02 × 10^−5^
**O3, O4, O5**		2.102, 2.482, 1.851							
N2, N4, N6,	19.82	2.497, 2.451, 2.111,	1564.3	1548.3	0.01280	1530.69	2.1905	1.128 × 10^6^	1.25 × 10^−5^
O2, O4, O6		2.153, 1.991, 1.94							
O10, O11, O12,	19.94	2.363, 2.326, 2.46,	1513.3	1542.8	0.01292	1531.70	2.3483	1.686 × 10^6^	1.99 × 10^−5^
O13, N13, N14		1.971, 2.512, 2.458							
O10, O11, O12,	20.68	2.360, 2.17, 2.085,	1485.6	1540.3	0.01343	1530.69	2.2930	1.316 × 10^6^	1.46 × 10^−5^
N12, N13, N14		2.396, 2.26, 2.487							
N11, N12, N13,	19.99	2.337, 2.012, 2.288,	1513.9	1543.0	0.01300	1529.68	2.2860	1.262 × 10^6^	1.44 × 10^−5^
N14, O10, O11		2.465, 2.157, 2.457							

**Table 4:** The atom #’s, dipole moment (Debye), bond distances (Å), area (Å^2^), volume (Å^3^), dipole/volume ratio (D/Å^3^), molar mass (g/mol), average bond length (Å), energy (joules), an aqueous stability factor of ten halichondrin B molecules containing iron.

Oxygen #’s	Dipole	Distance	Area	Volume	D/V	Molar mass	Avg. bond distance	Energy	ASF

**1,2,3,5,6,7**	13.87	2.639, 2.647, 2.574, 2.453, 2.684, 2.706	945.71	1060.93	0.01307	1167.57	2.617	3674624.	6.9 × 10^−5^
**3,5,6,7,9,10**	19.67	2.540, 2.488, 2.664, 2.710, 2.697, 2.727	1030.39	1080.05	0.01821	1167.57	2.194	7393466.	8.0 × 10^−5^
**1,3,5,6,7,9**	15.91	2.682, 2.540, 2.462, 2.695, 2.710, 2.683	941.03	1069.44	0.09596	1167.57	2.629	6085918.	1.0 × 10^−4^
**1,2,3,6,7,10**	19.94	2.717, 2.718, 2.664, 2.730, 2.680, 2.686	893.96	1051.35	0.01897	1167.57	2.699	−4685181.	−6.3 × 10^−5^
**2,5,7,9,10,12**	7.55	2.729, 2.769, 2.750, 2.782, 2.592, 2.729	930.78	1057.96	0.00714	1167.57	2.720	6953.642	2.5 × 10^−7^
**1,3,5,6,7,10**	18.78	2.673, 2.528, 2.526, 2.725, 2.728	1016.45	1076.59	0.01744	1167.57	2.649	7292712.	1.0 × 10^−4^
**2,3,5,6,7,14**	8.60	2.724, 2.482, 2.586, 2.710, 2.719, 2.772	887.65	1046.50	0.00822	1167.57	2.666	1394772.	4.3 × 10^−5^
**14,15,16,17,18,19**	11.75	2.687, 1.886, 2.614, 2.318, 2.219, 1.999	1027.22	1076.81	0.01091	1166.57	2.287	2559699.	5.0 × 10^−5^
**1,14,16,17,18,19**	11.31	2.634, 2.692, 2.699, 2.401, 2.084, 1.946	976.65	1065.90	0.01061	1166.60	2.416	3294402.	7.0 × 10^−5^
**1,15,16,17,18,19**	10.84	2.613,2.502, 2.616, 2.103, 2.255, 1.984	991.43	1071.33	0.01012	1166.57	2.059	3577592.	6.8 × 10^−5^

**Table 5:** The dipole moment (Debye), bond distances (Å), area (Å^2^), volume (Å^3^), dipole/volume ratio (D/Å^3^), molar mass (g/mol), average bond length (Å), energy (joules/mol), and aqueous stability factor (ASF) of ten Discodermolide molecules containing iron bound to water.

Oxygen-nitrogen #’s	Dipole	Distance	Area	Volume	D/V	Molar mass	Aver. Bond Dist.	Energy	ASF

**H2O, O2, O3, O6, O8, N1**	14.47	2.559, 2.091, 2.051, 2.237, 2.038, 2.118	704.04	674.36	0.0215	666.656	2.1823	787597.4	1.1878 × 10^−5^
**O1, O2, O3, H2O, O8, N1**	13.73	2.003, 2.122, 2.009, 2.547, 2.094, 1.995	706.77	675.37	0.0203	667.664	2.1283	685588.1	1.0628 × 10^−5^
**H2O, O2, O3, O4, O5, O6**	13.57	2.554, 2.350, 2.141, 2.446, 2.203, 2.213	690.23	669.93	0.0203	664.640	2.3178	814118.9	1.3906 × 10^−5^
**O1, O2, O3, H2O, O5, O6**	11.64	2.237, 2.217, 2.001, 2.530, 2.111, 2.117	692.78	669.60	0.0174	665.648	2.2022	614768.0	1.1631 × 10^−5^
**O1, O2, O3, H2O, O5, O8**	26.10	2.049,2.085, 2.018, 2.537, 2.211, 2.198	699.71	675.36	0.0386	667.664	2.1830	820896.2	6.8660 × 10^−6^
**H2O, O2, O3, O6, O7, N1**	27.13	2.532, 2.071, 2.132, 2.010, 2.392, 2.063	699.23	670.70	0.0405	665.648	2.2000	1155916.	9.3734 × 10^−6^
**O1, O2, O3, O4, O8, H2O**	21.48	1.933, 2.434, 1.962, 2.366, 2.267, 2.519	712.92	676.72	0.0317	667.664	2.2468	1109704.3	1.1608 × 10^−5^
**H2O, O3, O4, O7, O8, N1**	13.61	2.524, 2.074, 2.055, 2.286, 2.047, 2.136	709.82	676.42	0.0201	667.664	2.1870	801772.3	1.2884 × 10^−5^
**O1, O3, O4, H2O, O8, N1**	14.10	2.015, 2.108, 2.011, 2.533, 1.981, 2.003	710.93	676.08	0.0209	667.664	2.1085	865660.4	1.2945 × 10^−5^
**H2O, O3, O4, O5, O7, O8,**	26.56	2.552, 2.390, 2.322, 2.244, 2.155, 2.100	696.62	677.37	0.0392	667.664	2.2938	1399399.8	1.2086 × 10^−5^

**Table 6:** The dipole moment (Debye), bond distances (Å), area (Å^2^), volume (Å^3^), dipole/volume ratio (D/Å^3^), molar mass (g/mol), average bond length (Å), energy (joules), and aqueous stability factor of ten Kahalalide F molecules containing iron bound to water.

Oxygen-nitrogen #’s	Dipole (Debye)	Distance	Area	Volume	D/V	Molar mass	Ave. bond length	Energy	ASF

**H2O, N1, N2,**	19.52	2.438, 2.332, 2.55,	1535.1	1564.71	0.012475	1549.71	2.2488	1.28 × 10^6^	1.48 × 10^−5^
**O5, O6, O7**		1.946, 2.225, 2.002							
**H2O, O6, O7,**	13.76	2.479, 2.121, 2.477,	1570.3	1565.01	0.008792	1548.71	2.3408	8.64 × 10^5^	1.47 × 10^−5^
**N1, N2, N3**		2.556, 2.446, 1.966							
**H2O, O2, O3,**	27.44	2.427, 2.373, 2.44,	1574.0	1566.24	0.017519	1548.71	2.3018	1.77 × 10^6^	1.48 × 10^−5^
**N4, N5, N6**		2.58, 1.906, 2.085							
**H2O, N4, N6,**	23.48	2.535, 2.147, 2.017,	1588.7	1566.91	0.014984	1549.71	2.1355	1.12 × 10^6^	1.02 × 10^−5^
**O2, O4, O6**		1.972, 2.147, 1.995							
**H2O, O4, O6**	15.97	2.424, 2.362, 2.437,	1576.2	1567.12	0.010190	1548.71	2.2847	8.52 × 10^5^	1.22 × 10^−5^
**N2, N4, N6**		2.56, 1.978, 1.947							
**H2O, O10, O12,**	23.74	2.379, 2.587, 2.135,	1558.6	1564.31	0.015170	1548.71	2.361	1.23 × 10^6^	1.22 × 10^−5^
**N12, N13, N14**		2.268, 2.297, 2.5							
**H2O, O10, O11,**	25.02	2.198, 2.082, 2.58,	1524.4	1562.13	0.016016	1548.71	2.3227	1.38 × 10^6^	1.28 × 10^−5^
**N12, N13, N14**		2.281, 2.253, 2.542							
**H2O, O10, N11,**	36.57	2.131, 2.588, 2.504,	1563.9	1565.28	0.023363	1547.70	2.4075	1.56 × 10^6^	1.02 × 10^−5^
**N12, N13, N14**		2.262, 2.405, 2.555							
**H2O, O10, O11,**	20.90	2.126, 1.923, 2.338,	1534.3	1561.66	0.013383	1548.71	2.3348	1.30 × 10^6^	1.45 × 10^−5^
**N11, N12, N14**		2.494, 2.602, 2.526							
**H2O, O10, O11,**	22.12	2.347, 1.958, 2.461,	1559.1	1532.94	0.014429	1548.71	2.3022	1.14 × 10^6^	1.19 × 10^−5^
**N11, N12, N13**		2.102, 2.377, 2.568							

**Table 7:** The dipole moment (Debye), bond distances (Å), area (Å^2^), volume (Å^3^), dipole/volume ratio (D/Å^3^), molar mass (g/mol), average bond length (Å), energy (joules), and aqueous stability factor of ten halichondrin B molecules containing iron bound to water.

Oxygen #’s	Dipole	Distance	Area	Volume	D/V	MW	Aver bond dist	Energy	ASF

**1,3,5,6,7,H2O**	16.96	Fe-O1: 2.638, 1.88, 2.472, 2.211, 2.582, 2.659	971.93	1085.69	0.0156	1167.57	2.407	3103.006	4.4 × 10^−8^
**2,3,6,7,10,H2O**	17.89	Fe-H_2_O: 2.557, 2.128, 1.948, 2.512, 2.394, 2.301	952.20	1080.52	0.0166	1185.57	2.307	−1780.230	−2.3 × 10^−8^
**1,2,3,7,10,H2O**	10.73	Fe-O1: 2.327, 2.455, 5.367, 2.59, 2.054, 2.498	982.86	1086.39	0.0099	1185.57	2.382	−2600.840	−5.8 × 10^−8^
**2,3,5,6,7,H2O**	16.77	Fe-O2: 2.498, 1.848, 1.993, 2.314, 2.453, 2.498	966.88	1082.15	0.0155	1118.57	2.270	−125.424	−1.7 × 10^−9^
**15,16,17,18,19, H2O**	14.25	Fe-H_2_O: 2.511, 2.535, 2.483, 2.151, 2.25, 1.997	1057.85	1095.69	0.0130	1185.57	2.320	2657.263	4.33 × 10^−8^
**14,16,17,18,19,H2O**	15.12	Fe-H_2_O: 2.492, 2.631, 2.618, 2.268, 2.154, 1.997	1047.75	1093.53	0.0138	1185.57	2.360	2512.387	3.92 × 10^−8^
**1,16,17,18,19,H2O**	13.27	Fe-O1: 2.572, 2.516, 2.533, 2.088, 2.386, 2.033	1020.44	1089.88	0.0122	1185.57	2.350	2201.572	3.9 × 10^−8^
**1,14,16,18,19,H2O**	19.50	Fe-O1: 2.574, 2.642, 2.701, 2.558, 2.104, 1.951	1009.10	1085.79	0.0178	1185.57	2.420	2320.397	2.88 × 10^−8^
**15,16,17,18,19,H2O**	16.02	Fe-H_2_O: 2.510, 2.538, 2.484, 2.149, 2.259, 2.00	1057.95	1095.55	0.0146	1185.57	2.323	2657.028	3.85 × 10^−8^
**1,16,17,18,19,H2O**	12.83	Fe-O1: 2.562, 1.955, 2.553, 2.239, 2.221, 2.017	1023.60	1090.34	0.0118	1185.57	2.258	2189.281	3.85 × 10^−8^

**Table 8:** The average values for approximately fifty complexes of each iron species.

Species	Area	Volume	Dipole Moment	D/V	ABL	Energy	ASF_Best	Average ASF
**Fe-Hali**	949.7	1065.0	8.82	0.0110	2.627	6307365	−0.0000634	**0.000253**
**Fe-Hali-H2O**	991.7	1087.2	13.83	0.0126	2.483	3946000	0.00000086	−**5.77E-08**
**Hali**	1036.5	1071.7	2.48	0.0023		1491027		
**Fe-Disco**	656.4	650.6	6.48	0.0100	2.231	1787000	0.0000153	**0.00389**
**Fe-Disco_H2O**	697.2	673.2	10.68	0.0159	2.211	1090000	0.00000686	**0.0000281**
**Disco**	689.9	650.9	4.84	0.0074		135515		
**Fe-Kah**	1537.0	1546.6	15.99	0.0103	2.283	2005200	0.00001246	**0.00156**
**Fe-Kah-H2O**	1565.9	1565.1	19.04	0.0121	2.325	1551000	0.00001988	**0.000003406**
**Kah**	1584.3	1548.0	4.82	0.0031		853007		
